# Difference in the recurrence rate between right- and left-sided colon cancer: a 17-year experience at a single institution

**DOI:** 10.1007/s00595-013-0748-5

**Published:** 2013-10-16

**Authors:** Konosuke Moritani, Hirotoshi Hasegawa, Koji Okabayashi, Yoshiyuki Ishii, Takashi Endo, Yuko Kitagawa

**Affiliations:** Department of Surgery, School of Medicine, Keio University, 35 Shinano-machi, Shinjuku-ku, Tokyo, Japan

**Keywords:** Colon carcinoma, Proximal colon, Distal colon, Prognosis, Recurrence

## Abstract

**Purpose:**

The prognostic differences between right- and left-sided colon cancer are controversial. This study aimed to clarify the clinical difference between right- and left-sided colon cancer.

**Methods:**

We enrolled 820 patients with stage I/II/III colon cancer who underwent radical surgery with curative intent. We explored the impact of the tumor location on the postoperative disease-free survival (DFS) rate using the univariate and multivariate analyses.

**Results:**

Right-sided disease occurred in 399 of the 820 patients. The mean follow-up period was 55.8 ± 34.9 months. The pathological stage distribution was as follows: stage I 261 patients; stage II 283; and stage III 251. There were no significant differences in the five-year DFS of the overall populations (right 88.6 %; left 89.4 %; *P* = 0.231). The subgroup analyses demonstrated that patients with stage I right-sided colon cancer had a significantly better 5-year DFS rate than did those with left-sided disease (100 vs. 95.2 %, *P* = 0.034). There were no significant differences in the distributions of the first recurrent sites (*P* = 0.559).

**Conclusions:**

The tumor location may contribute to postoperative tumor recurrence. However, these effects were inconsistent across tumor stages. Our results provide a better understanding of the prognostic disparity between tumor locations; this may improve patient consent and postoperative surveillance.

## Introduction

Colon cancer is one of the most common malignancies worldwide, and despite improvements in the treatments for locally advanced and metastatic disease, colon cancer is the third leading cause of cancer-related deaths in Japan. Vital Statistics Japan (Ministry of Health, Labour and Welfare) estimated that 42,800 deaths were attributable to colon cancer in Japan in 2009.

Based on the incipient differences between right- and left-sided colon cancer, the relevance of the tumor location in the postoperative prognosis of colon cancer has been explored for a few decades. Several single-institutional cohort studies published in the 1980s concluded that the tumor location had no impact on the overall survival [1, 2], whereas recent nationwide cohort studies demonstrated that right-sided colon cancer was associated with a worse overall survival than was left-sided colon cancer [3–7]. A better understanding of the site-specific survival outcomes would be helpful for guiding research and education, but the influence of the tumor location on the survival outcomes remains unclear.

The impact of the tumor stage and location on the survival outcomes is also imprecise. A large American retrospective cohort study, namely, the surveillance, epidemiology, and end results (SEER) program, showed that patients with stage II right-sided colon cancer had a significantly lower hazard ratio (HR) than did patients with left-sided colon cancers; patients with stage III and IV right-sided colon cancer had a significantly higher HR than did patients with the same stage of left-sided colon cancer [6]. To understand these distinctive results, it is important to identify the patterns of recurrence according to the tumor stage and location. However, only one study has so far examined the patterns of postoperative recurrence by tumor location, reporting systemic and local recurrence without describing the organs affected by the colon cancer metastasis [4].

The aim of the present study was to examine the differences in oncological behavior according to the tumor location in colon cancer, using a single institutional Japanese database without racial diversity. The results will be helpful to many surgeons for developing treatment strategies and in the patient consent process.

## Methods

**Fig. 1:**
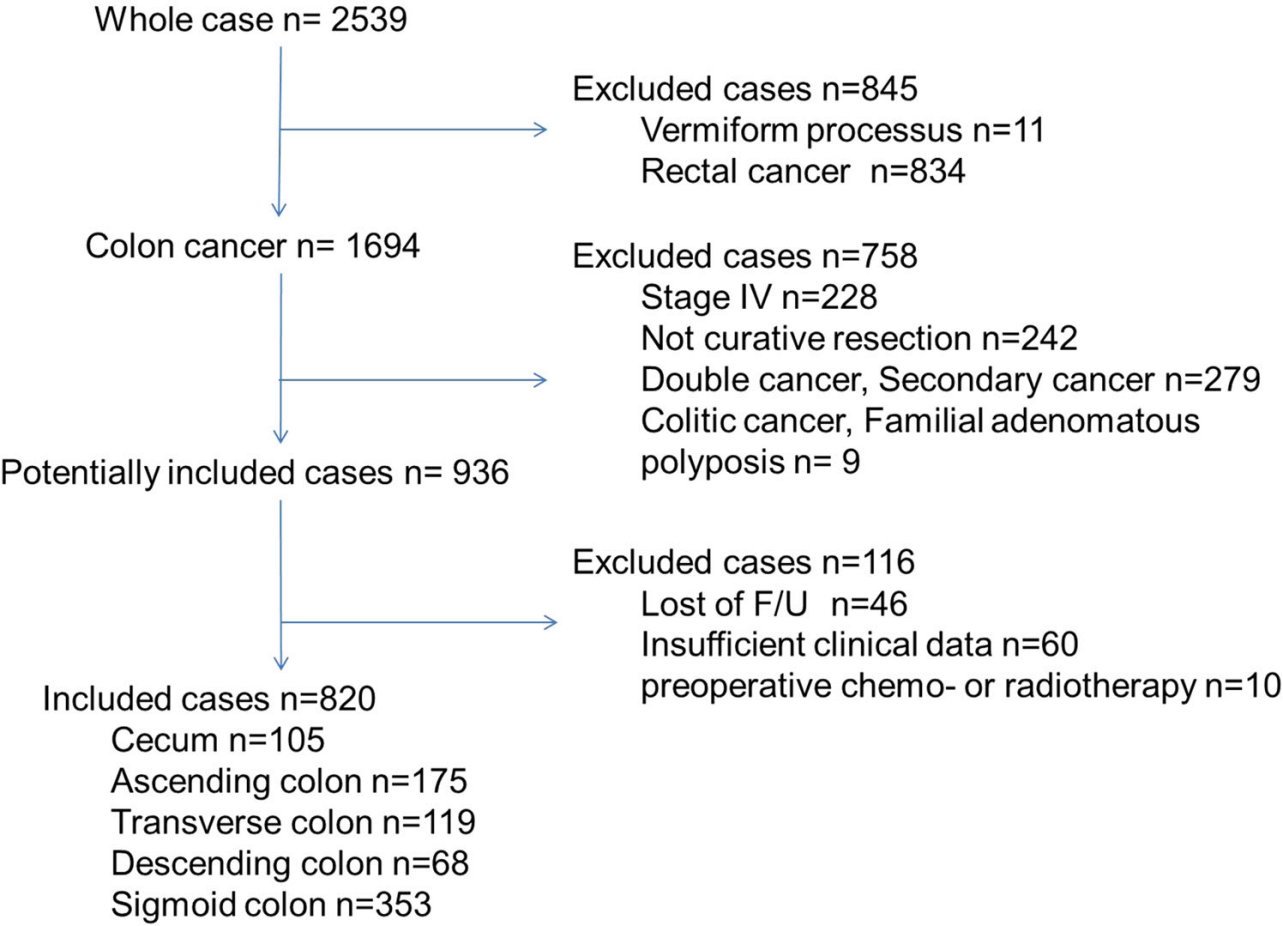
A flow chart of the patient inclusions between January 1990 and January 2006

The procedures used for including patients are shown in Fig. 1. Between January 1990 and January 2006, 1,694 patients with colon cancer were treated surgically at our institute; 820 underwent radical surgery with curative intent for colon cancer, and these formed the basis of our study. Histopathological staging was confirmed postoperatively by a consulting pathologist through examinations of retrieved specimens.

The tumor location from the cecum to the sigmoid colon was classified by the TNM criteria 6th edition. The cecum and the ascending and transverse colon were defined as the right-sided colon, whereas the descending and sigmoid colon were defined as the left-sided colon. We excluded patients receiving preoperative chemo- or radiotherapy, which can affect the postoperative survival.

The demographic variables examined included the age, sex, year of diagnosis, stage, histological grade, and number of lymph nodes examined in the surgical specimen. Survival data were confirmed at every hospital visit or by telephone.

This study was approved by the ethics review board in our institute.

### Surveillance program

All patients were followed up according to our scheduled surveillance program, which mainly consisted of physical examination, measurement of the serum tumor markers (carcinoembryonic antigen (CEA) and carbohydrate antigen 19-9 [CA 19-9]), computed tomography, and colonoscopy. Patients with stage I cancer were followed up every 6 months with physical examinations, measurement of the serum tumor marker levels (CEA and CA 19-9) and computed tomography. For patients with stage II and III cancer, the serum tumor marker levels were examined every 3 months and computed tomography was performed every 6 months for up to 5 years. Colonoscopy was performed 1 year after the operation and every 3 years thereafter for all patients. Postoperative surveillance was usually discontinued for patients without any relapse at 6 years after surgery, but it was continued in this case at the request of the patient.

### Statistical methods

The continuous data were analyzed using Student’s *t* test. Differences in continuous variables were compared by testing the differences in the medians by the Kruskal–Wallis test. The survival analysis was performed by the Kaplan–Meier method, and the log-rank test was used to determine the statistical significance of differences. Cox proportional hazard methods were used to assess the multivariate predictors of outcomes. All statistical tests were two-sided, and a value of *P* *=* 0.05 was considered to be statistically significant. The statistical analyses were performed using the SPSS software program (version 17, SPSS, Chicago, Illinois).

## Results

### Patient characteristics

The patient characteristics are shown in Table 1. Right-sided disease was present in 49 % (399/820) of the included patients. The mean patient age was 64.2 ± 11.6 years; 497 participants (60.6 %) were males. The pathological stage distribution was as follows: 261 individuals had stage I disease; 283, stage II disease; and 251, stage III disease. The patients with right-sided colon cancers were significantly older (right-sided 65.9 ± 11.2 years; left-sided 62.2 ± 11.8; *P* < 0.001) and exhibited a poorer performance status (0/1/2/3/4; right-sided, 224/88/14/3/2 vs. left-sided, 264/72/6/5/0; *P* = 0.032), than those with left-sided disease. There was a significant difference in the length of the operation between the groups (right-sided 175.6 min vs. left-sided 187.3 min; *P* = 0.023). Moreover, the primary tumors differed significantly in terms of the proportion of poorly differentiated adenocarcinoma (right-sided 26/386 vs. left-sided 11/413; *P* = 0.011), tumor size (right-sided 4.6 ± 2.7 cm vs. left-sided 3.8 ± 2.0; *P* < 0.001), and tumor stage (stage I/II/III; right-sided, 110/147/131 vs. left-sided, 151/136/120; *P* = 0.032). The mean number of retrieved lymph nodes was 21.8 in cases of right-sided colon cancer and 16.4 in cases of left-sided disease (*P* < 0.001). There was no significant difference in the postoperative use of adjuvant chemotherapy (Chi square test*, P* = 0.631) (Table 2).

**Table 1:** The patient backgrounds

Variables		Right	Left	Total	*P* value
Number of patients		399	421	820	
Baseline variables					
Age	Mean ± SD	65.97 ± 11.3	62.23 ± 11.9	64.25 ± 11.6	<0.001
Gender	Male/female	250/149	247/174	497/323	0.243
Body mass index	Mean ± SD	22.91 ± 3.6	22.36 ± 3.3	22.63 ± 3.6	0.052
Performance status	0	224	264	488	0.037
	1/2	88/14	72/6	160/20	
	3/4	3/2	5/0	8/2	
Family history	Yes/no	38/340	43/362	81/702	0.796
Surgery-related variables					
Length of operation	Mean ± SD	175.6 ± 78.0	187.3 ± 78.0	182.5 ± 76.8	0.023
Estimated blood loss	Mean ± SD	144.4 ± 217.7	146.9236.5	140.8 ± 218.8	0.955
Emergency operation	Yes/no	3/391	5/412	8/803	0.726^a^
Laparoscopic surgery	Yes/no	152/243	176/244	328/487	0.319
Pathological variables					
Tumor size	Mean ± SD	4.6 ± 2.7	3.8 ± 2.0	4.2 ± 2.4	<0.001
Pathological grade	wel/mod	360	402	762	0.011
	por/muc/sig	26	11	37	
	Others	5	2	7	
TNM stage	I	110	151	261	0.032
	II	147	136	283	
	III	131	120	251	
Number of harvested LNs	Mean ± SD	21.8 ± 13.1	16.4 ± 11.3	19.3 ± 12.5	<0.001
Number of metastatic LNs	Mean ± SD	1.2 ± 2.7	0.8 ± 1.8	0.96 ± 2.2	0.027
Use of adjuvant chemotherapy	Yes/no	115/284	115/306		0.631^b^

*SD* standard deviation, *wel* well-differentiated tubular adenocarcinoma, *mod* moderately differentiated tubular adenocarcinoma, *por* poorly differentiated tubular adenocarcinoma, *muc* mucinous adenocarcinoma, *sig* signet-ring cell adenocarcinoma, *LN* lymph node

^a^Fisher’s exact test

^b^Chi square test

**Table 2:** The patient backgrounds stratified by stage

Variables		Stage I			Stage II/III		
		Right (*n* = 110)	Left (*n* = 151)	*P* value	Right (*n* = 278)	Left (*n* = 256)	*P* value
Baseline variables							
Age	Mean +/SD	64.8 ± 10.8	61.4 ± 11.3	0.014	66.4 ± 11.7	62.6 ± 12.0	0.003
Gender	Male/female	77/33	55/96	0.278	168/110	144/115	0.242
Body mass index	Mean ± SD	23.1 ± 3.2	22.9 ± 3.2	0.630	22.9 ± 3.7	22.1 ± 3.3	0.055
Performance status	0	70	106	0.206	151	154	0.159
	1/2	19/2	24/0		64/12	44/6	
	3/4	0/0	0/0		3/2	4/0	
Family history	Yes/no	14/90	15/136	0.450	22/244	26/244	0.405
Pathological variable							
Tumor size	Mean ± SD	2.8 ± 2.5	2.2 ± 1.2	0.008	5.1 ± 2.5	4.6 ± 1.9	0.001
Pathological grade	wel/mod	105	144	0.483	251	250	0.006
	por/muc/sig	2	3		24	8	
	Others	0	2		3	0	
Number of harvested LNs	Mean ± SD	16.6 ± 12.4	11.7 ± 11.2	0.001	23.9 ± 12.8	19.5 ± 10.1	<0.001
Number of metastatic LNs	Mean ± SD	–	–	–	1.6 ± 3.1	1.2 ± 2.1	0.069

*SD* standard deviation, *wel* well-differentiated tubular adenocarcinoma, *mod* moderately differentiated tubular adenocarcinoma, *por* poorly differentiated tubular adenocarcinoma, *muc* mucinous adenocarcinoma, *sig* signet-ring cell adenocarcinoma, *LN* lymph node

### Difference in the DFS of colon cancer based on the tumor location

**Fig. 2:**
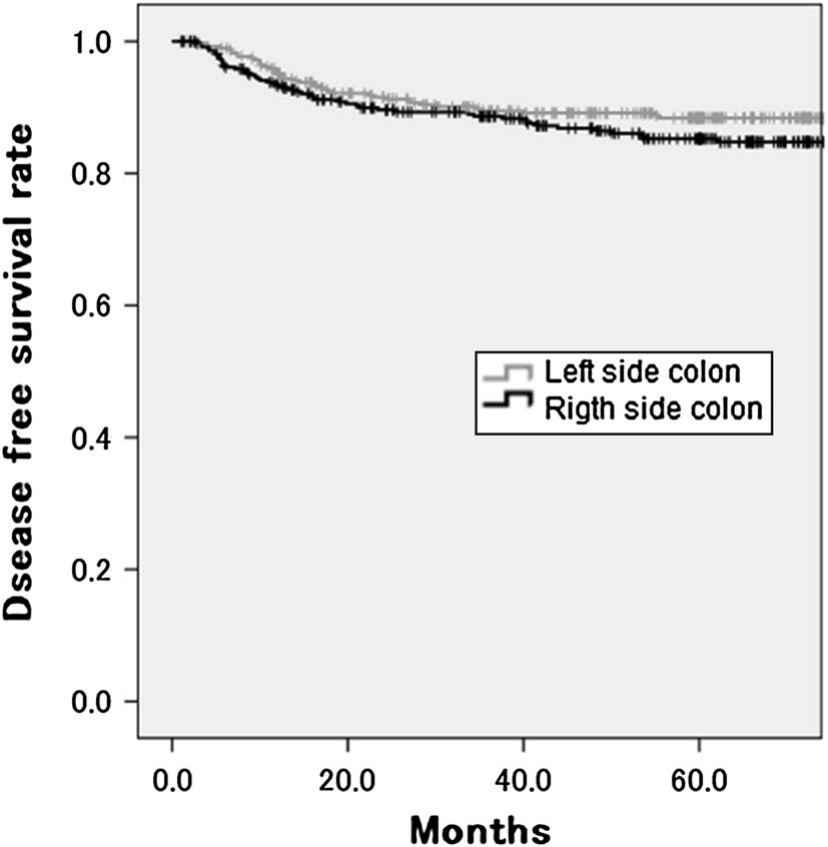
The five-year disease-free survival rate of patients with right- and left-sided colon cancer. There was no significant difference between right- and left-sided disease (right 85.2 %; left 88.4 %; *P* = 0.231)

The mean follow-up period in the overall population of patients was 55.8 ± 34.9 months; the three-year and five-year DFS rates were 88.6 and 85.2 % in right-sided colon cancer patients and 89.4 and 88.4 % in left-sided colon cancer patients, respectively. There was no significant difference between the two groups (log-rank, *P* = 0.231; Fig. 2). Compared to left-sided colon cancer, the unadjusted hazard ratio (HR) for right-sided colon cancer was estimated to be 1.253 (95 % confidence interval (CI), 0.837–1.874), while the HR adjusted by age, performance status, size, and TNM stage was estimated to be 0.948 (95 % CI, 0.592–1.518). Moreover, the adjusted HRs were estimated to be 1.096 (95 % CI, 0.657–1.827) for cecum or ascending colon cancer, 0.760 (95 % CI, 0.330–1.749) for transverse colon cancer, and 1.152 (95 % CI, 0.476–2.785) for descending colon cancer (Table 3).

**Table 3:** The results of the multivariate survival analysis based on the tumor location

Colon site	Hazard ratio^a^	95 % CI	*P* value
Cecum and ascending colon	1.096	0.657–1.827	0.148
Transverse colon	0.760	0.330–1.749	0.547
Descending colon	1.152	0.476–2.785	0.540
Sigmoid colon	1		

^a^Hazard ratio (HR) adjusted by age, performance status, size and TNM stage

**Fig. 3:**
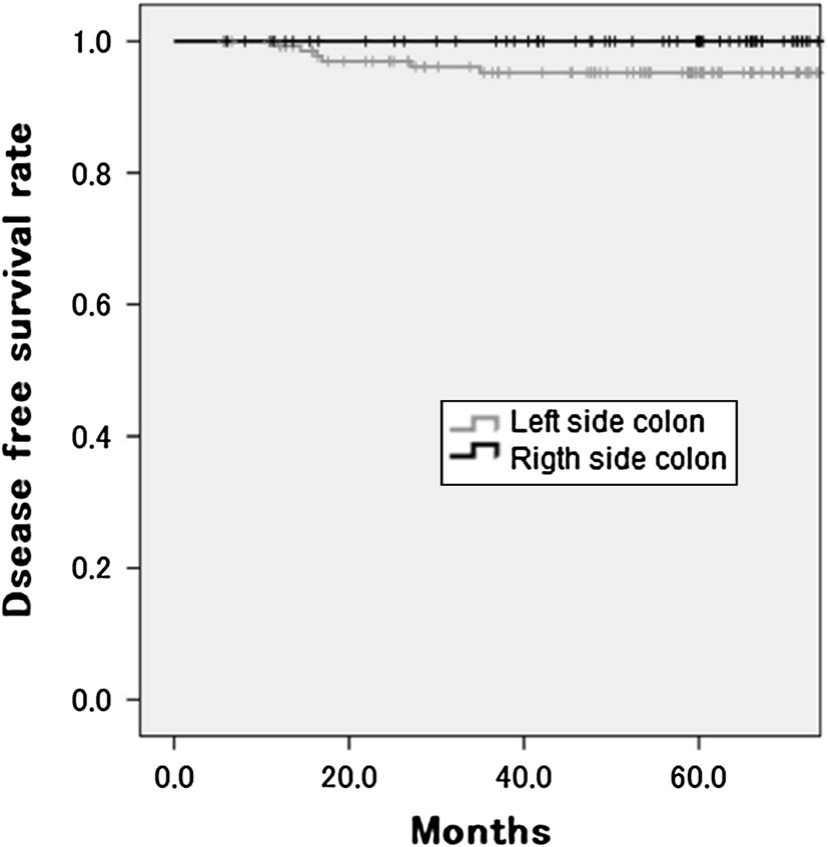
The five-year disease-free survival rate of patients with right- and left-sided colon cancer in stage I. Patients with right-sided colon cancer had a significantly better survival rate (100 vs. 95.2 %, *P* = 0.034)

**Fig. 4:**
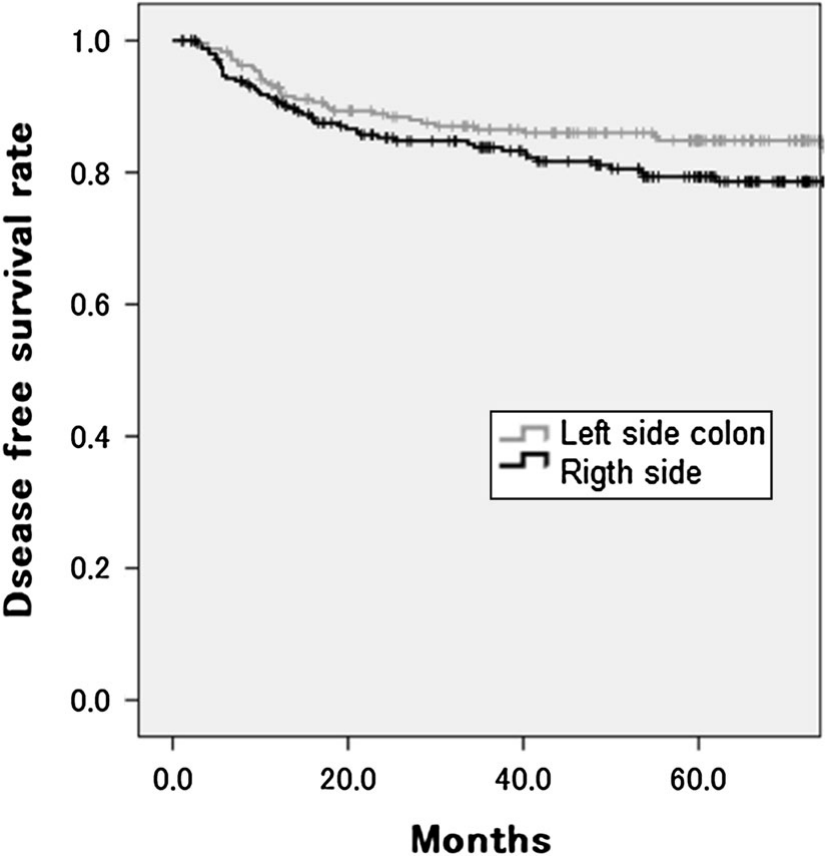
The five-year disease-free survival rate of patients with right- and left-sided colon cancer in stages II/III. There was no significant difference between right- and left-sided disease (right 79.4 %; left 84.7 %; *P* = 0.152)

We added the subgroup analyses to assess the influence of differences in the baseline characteristics. The subgroup analyses demonstrated that patients with right-sided colon cancer had a significantly better five-year DFS than did the patients with left-sided disease (100 vs. 95.2 %, *P* = 0.034) at stage I. However, there was no significant difference at stages II and III (right-sided 79.4 %; left-sided 84.7 %, *P* = 0.152; Figs. 3, 4).

### Details of recurrence and treatment after recurrence

Postoperative recurrence developed in 94 patients, 50 of whom had right-sided colon cancer. The details of the recurrent sites by each tumor location are shown in Table 4. There were no significant differences in the distribution of the first recurrent sites between tumor locations (Chi square test, *P* = 0.559). The treatment after recurrence included salvage surgery in 26 (27.7 %) patients and systemic chemotherapy in 22 (23.4 %); there was no significant difference in the treatment after recurrence between the tumor locations (Chi square test, *P* = 0.753).

**Table 4:** The site of recurrence of right- and left-sided colon cancer

Variables	Right	Left	Total	*P* value
Number of patients with recurrence	51	39	90	0.272
Site of recurrence				
Local	2 (4 %)	1 (3 %)	3	0.753
Liver	20 (39 %)	20 (51 %)	40	
Lung	13 (25 %)	8 (21 %)	21	
Bone	1 (2 %)	2 (5 %)	3	
Disseminated	1 (2 %)	1 (3 %)	2	
Others	12 (24 %)	7 (18 %)	19	
Resection rate (%)	25.4	33.3	28.9	0.625

## Discussion

Our data show that right-sided colon cancer had a marginally worse DFS rate than left-sided cancer. This association between the tumor location and DFS was especially clear in advanced colon cancer (stage II or III), but was inconsistent for patients with stage I disease. Furthermore, there was no significant difference in the distribution of the first recurrence site between the patients with right- and left-sided disease.

Although the overall survival rate was defined as a primary endpoint in previous studies assessing the prognostic difference between tumor locations, we used the DFS rate as a primary endpoint for two reasons. First, the DFS rate, which is calculated based on tumor recurrence, describes the metastatic potential more precisely than does the overall survival rate. Second, there is a paucity of evidence regarding the patterns of recurrence based on tumor location. Our results on the DFS rate lead to a better understanding of the prognostic differences between right-sided and left-sided colon cancer, and can improve patient management.

One large cohort study reported that the prognostic disparity according to tumor location was inconsistent across tumor stages, similar to our results [6]. Although the reasons for this disparity are unclear, some oncological data may be pertinent. Advanced right-sided colon cancers were more significantly associated with poorly differentiated adenocarcinoma than were left-sided cancers. This differential prevalence of poorly differentiated adenocarcinoma may contribute to the worse prognosis of right-sided advanced colon cancer. In addition, the negative association between right-sided colon cancer and recurrence disappeared in early stage colon cancers. This suggests that right-sided colon cancers acquire worse oncological potential in late stages of tumor development. Furthermore, tumors with microsatellite instability, which more frequently develops in right-sided colon cancer, have been associated with better prognosis; this may explain the reduction of recurrence [8–10]. A better understanding of these disparate results will require the use of a prospective database with a large number of patients and including more clinical and pathological details.

During embryologic development, the right colon arises from the midgut and the left colon from the hindgut. The right and left colons are exposed to different luminal contents. Consequently, many studies have been conducted to explore these theoretical genetic backgrounds of the different lesions. Although *BRAF* mutations were reported to be a risk factor for metastatic spread, peritoneal metastases, and distant lymph node metastases [11], *BRAF* mutations were observed more frequently in the proximal colon [12]. In addition, the genome-wide analyses using primary tumor specimens have revealed differential gene expression patterns in both tumor locations. These studies may be useful for identifying prognostic differences, but future investigations addressing prognostic biomarkers are required to acquire robust data and to improve the postoperative surveillance and treatment.

There are some differences between Japanese and Western colorectal cancer (CRC) screening systems. In Japan, although the approach to CRC screening remains centered on the use of fecal immunochemical testing, most patients diagnosed with CRC are recommended to undergo total colonoscopy. On the other hand, in the US, there has been a shift toward a more complex approach to CRC screening, in which options such as fecal occult blood testing and colonoscopy are regularly employed. However, US experts did not recommend colonoscopy for CRC screening until 2002, and sigmoid scope examinations were mainly performed for patients diagnosed with CRC [13, 14]. The percentage of early disease detected in the right colon increased significantly, from 22.1 to 24.1 %, after Medicare approved colonoscopy for CRC screening, indicating that colonoscopy improved the early detection of proximal tumors [15]. Clarification of the influence of nation health systems on cancer-related mortality requires future investigation of global cancer statistics and health systems. These reasons may also explain why the rate of stage I right-sided colon cancer was high, at 27.5 %, in our study.

Most previous studies have focused on Western populations and demonstrated no racial disparity in the tumor recurrence or cancer-related death based on the tumor location. However, there is a paucity of data concerning the relationship between the tumor location and survival outcomes in Asian patients. A recent South Korean study found that left-sided colon cancer was a risk factor for recurrence as well as the preoperative CEA level, T-stage, N-stage, and use of postoperative chemotherapy in a multivariate analysis, in contrast to the Western data and our present results [16]. Another study reported that Asian patients had better overall survival than did non-Hispanic white and black patients [5]. However, these analyses were performed based on a database established in Western countries and containing a small number of enrolled cases of racial minorities [17]. Further investigations of non-Western populations are required to explore the impacts of the tumor location on the prognostic disparity.

There are some limitations to our study that should be kept in mind when interpreting the results. Although most previous studies assessed similar outcomes using a nationwide database [3–7], the statistical power of our study is somewhat weak due to the retrospective nature of the analysis, the fact that the data were from a single institution and the relatively small sample size included in the analysis. Moreover, despite the availability of modern powerful chemotherapy for CRC patients [18, 19], the impact of adjuvant chemotherapy on CRC has not been evaluated, leading to significant heterogeneity resulting from changing regimens. The patients analyzed in this study have been collected retrospectively over 17 years, and during this time the health care environment with regard to the treatment of colorectal cancer patients, including the use of postoperative chemotherapy, has changed a lot. This study showed that there was no significant difference in the use of postoperative adjuvant chemotherapy between the groups; however, it may be better to evaluate the status of postoperative chemotherapy and chemotherapy for recurrent cancer separately. Although one previous study reported that the incidence of synchronous liver metastasis differs between proximal and distal colon cancer, there were no significant differences in the distribution of the first recurrence site between right- and left-sided diseases in our data [20].

Another limitation of this study is the failure to determine the cause(s) of the observed disparity, because an exploratory data analysis (perhaps leading to identification of a crucial gene) was not performed.

In conclusion, compared to left-sided colon cancer, right-sided colon cancer is a marginally significant risk factor for recurrence in patients with advanced colon cancer (stage II or III). In contrast, right-sided colon cancer at stage I has a significantly better prognosis. Furthermore, there are no differences in the distribution of the first recurrent site based on the primary tumor location. Our results provide a better understanding of the prognostic disparity between tumor locations; this may improve patient consent and postoperative surveillance.
